# Researches on Transcriptome Sequencing in the Study of Traditional Chinese Medicine

**DOI:** 10.1155/2017/7521363

**Published:** 2017-08-16

**Authors:** Jie Xin, Rong-chao Zhang, Lei Wang, Yong-qing Zhang

**Affiliations:** School of Pharmacy, Shan Dong University of Traditional Chinese Medicine, Jinan 250355, China

## Abstract

Due to its incomparable advantages, the application of transcriptome sequencing in the study of traditional Chinese medicine attracts more and more attention of researchers, which greatly promote the development of traditional Chinese medicine. In this paper, the applications of transcriptome sequencing in traditional Chinese medicine were summarized by reviewing recent related papers.

## 1. Introduction

As an important part of human brilliant culture, Chinese medicine has made an important contribution to the survival and reproduction of mankind. In the modern disease prevention and health care model, the traditional Chinese medicine has great superiority for a thousand years of accumulation of precipitation. In recent years, the transcriptome studies developed rapidly. In animal studies, there are a lot of reports on the discovery of key genes, SNP, gene splicing, and complex disease research [1, 2]. The plant transcription study also showed great application potential. For example, Wang et al. (2010) using Solexa sequencing technology constructed the transcriptome and the expression profiling of fuzzless-lintless mutant and screened the key genes involved in control of cotton fiber development [3]. Zhou et al. (2011) obtained 7,155 sequences from the cauliflower and screened out 1,600 genes related to HYPOCOTYL5 (HY5) under the light [4]. He et al. (2012) got 1,244,501 transcripts from the reeds and selected 251 genes related to the plant allelopathy, development, and invasion, providing valuable information for stress regulation mechanism and the nonmodel plant development [5]. In addition, large scale studies on the medicinal plants have also been carried out [6, 7].

The transcriptome sequencing technology has experienced three generations change. At present, the second-generation sequencing technology still dominates; the third-generation sequencing technology shows a strong momentum of development. The characteristics comparison of three-generation sequencing technology is shown in Table 1. Now, the deep sequencing technology is able to identify all the transcripts, even lacking detailed genetic information or with no reference genome [8]. As a whole research on gene expression, transcriptome studies changed the single gene study pattern and brought gene expression regulation and genome research into a rapid development era [9]. This paper reviewed the main applications of transcriptome sequencing in the study of traditional Chinese medicine, which provided references for the further study and development of Chinese medicine.

## 2. The Application of Transcriptome in Chinese Medicine

### 2.1. Construction of Gene Library (cDNA) and Transcription Database

Due to lack of genomic data, gene sequence information, and genetic background, the medical plants need a large amount of genetic information in order to analyze the gene function in the whole level. At present, the whole genome sequencing of most medicinal plants cannot be detected, so it is a quick way to compare the gene sequences, discover new genes, and identify the expressed genes by constructing the genomic library (cDNA) or the transcription database.

Large scale transcriptome studies have been carried out both in China and abroad on the medicinal plants such as American ginseng [7, 10, 11], liquorice [12], and* Salvia miltiorrhiza* [6]. Wildung and Croteau (1996) identified one key enzyme gene in the biosynthesis of taxol by using a homology probe to screen the cDNA library of Pacific ginseng combined with GC-MS method [13]. Ginseng (*Panax ginseng* C. A. Mey.) transcriptome (root, stem, leaf, and flower were sequenced, respectively) obtained 178,145 unique genes where 105,522 genes were discovered for the first time, and 65.6% of the newly independent genes were found in the stems, leaves, and flowers library [14]. Astragalus (*Astragalus membranaceus* (Fisch.) Bunge) transcriptome (root, stem, and leaf equivalent amount of RNA mixed sample) was successfully annotated with 9,732 Unigenes after de novo sequencing, and 2,877 of them were classified into 45 metabolic pathways by KEGG [15].

Up to March 2017, more than 50 species of traditional Chinese herbal medicine have been sequenced. Especially since 2014, the transcriptome study of traditional Chinese medicine developed rapidly. We have carried on the statistics to the common traditional Chinese medicine; specific sequence statistics are shown in Table 2.

### 2.2. Identification of the Enzymes Coding Genes Involved in Biosynthetic Pathway of Secondary Metabolites

Secondary metabolites are usually effective constituents or regulatory substances in Chinese medicine. The secondary metabolites include glycosides, alkaloids, saponins, flavonoids, steroids, organic acids, and many key enzymes in metabolic process. The mechanism of “multicomponent and multitarget” has made the secondary metabolites and its regulation mechanism become one of the important contents in the modern Chinese medicine research. Secondary, not only do metabolites play an important role in plants adapting to the special ecological environment and resistance to biotic or abiotic stresses (especially some enzymes), but many secondary metabolites with special biological activity are important active components of medicinal plants. The description of transcriptome can provide new information about biology and biochemistry, which can be used to find synthesis genes and its expression pattern of secondary metabolites and finally to determine its biosynthetic pathway. Because most of transcriptome studies in medicinal plants focus on the biosynthesis of active compounds and the research about this part is so much, this part is summarized as chemical composition.

Study on flavonoids related genes and enzymes:* Lepidium* seed is commonly used in traditional Chinese medicine where a variety of medicinal components have been extracted, but the synthesis genetic basis of these substances is still not clear.* Lepidium* seed transcriptome sequencing found 534 genes involved in the synthesis and metabolism of secondary metabolites. The genes in the biosynthetic pathway of mustard glycosides, flavonoids, and glycosides compounds were, respectively, 4, 19, and 69 Unigenes and 92 Unigenes in the phenylalanine metabolic pathway [16]. Huang et al. (2012) used KEGG to predict the biosynthetic pathways of flavonoids and unsaturated fatty acids in safflower (*Carthamus tinctorius *L.) and showed that the genes related to the pathway were conserved [17]. Wang et al. (2017) studied C-glycosyltransferases (CGTs) effect to the synthesis of isoflavonoids and revealed that PlUGT43, a novel glucosyltransferase, possesses an activity for the C-glycosylation of daidzein to puerarin [18].

Study on alkaloids related genes and enzymes:* Dendrobium officinale* L. is an important traditional herb with high commercial value and excellent medicinal effect in China. Li et al. (2017) analyzed the genes involved in dendrobine biosynthesis in* Dendrobium nobile* Lindl., and they found 30 Unigenes encoding proteins were possibly related to the biosynthesis of dendrobine sesquiterpene backbone; MF23 might stimulate dendrobine biosynthesis by regulating the expressions of genes involved in the mevalonate (MVA) pathway and postmodification enzymes might play a major role in dendrobine biosynthesis [19]. Shen et al. (2017) also identified the genes associated with the synthesis of bioactive constituents in* Dendrobium officinale*, which got Unigenes related to the enzymes involved in fructose and mannose metabolism and Unigenes associated with putative upstream elements of the alkaloid biosynthetic pathway [20]. Iron* Dendrobium* transcription sequencing data found that 25 enzyme genes were involved in alkaloid synthesis [14].

Study on terpenes and glycosides related genes and enzymes: the roots of* Platycodon grandiflorus* are widely used as a crude drug. The active components include a variety of triterpenoid saponins. Tamura et al. (2017) varied cytochrome P450 monooxygenases (P450s) function in triterpenoid saponin biosynthesis of* Platycodon grandiflorus* by the analysis of three different tissues (roots, leaves, and petals) using RNA sequencing (RNA-Seq) technology [21]. Ma et al. (2016) and Liu et al. (2017) analyzed the transcriptome of* Swertia mussotii* and screened 39 candidate transcripts involved in secoiridoid biosynthesis [22]. Transcriptional regulation of picrosides biosynthesis in* Picrorhiza kurroa* is completely unknown until Vashisht et al. (2016) investigated complete spectrum of different transcription factors and discerned their association with picrosides biosynthesis [23]. Wu et al. (2010) obtained the specific gene expression profiles and found 24 genes that might be involved in ginsenoside biosynthesis by analyzing the American ginseng transcription of 3 different tissues. And a number of transcription factors genes involved in the regulation of growth and response to environmental stress were obtained by sequence comparison and functional annotation [11]. Wu et al. (2012) obtained 23,532 transcripts from the foxglove, screened 140 key genes involved in the biosynthesis of cardiac glycosides, and identified 29 new miRNAs [24]. In addition,* Camptotheca acuminata* transcriptome acquired 20 genes involved in the synthesis of camptothecin skeleton, including 13 new genes [25].

Study on organic acids related genes and enzymes: Li et al. (2017) by analyzing 18 libraries from six organs, namely, roots, stems, leaves, sepals, flowers, and seeds of* D. Tanguticum*, found 22 predicted biosynthetic genes related to RA and two of these genes were identified as candidates by evaluating the correlation coefficient between the RA contents and the expression of the predicted biosynthetic genes in the six organs [61]. Li et al. (2010) by using high throughput sequencing technology and bioinformatics analysis obtained more than 27,000 Unigenes and 16 enzymes genes in the biosynthetic pathway of the glycyrrhizic acid [12]. Honeysuckle flower and leaf EST library found that almost all enzymes participate in the chlorogenic acid and luteolin biosynthetic and established the chlorogenic acid and luteolin biosynthetic pathway. The synthesis of* Ganoderma* triterpenoids is regulated by 11 upstream pathway key enzymes and 214 candidate cytochrome oxidase genes of downstream biosynthetic pathway [66].* Lonicera japonica* is one of the most important medicinal plants. Chlorogenic acid, luteolin, other flavonoids, and secoiridoids are the most important effective ingredients. Rai et al. (2016) studied the potential candidate genes involved in chlorogenic acid, luteolosides, and secoiridoid biosynthesis pathways of nine tissues of* Lonicera japonica* and the results showed that different tissues of* L. japonica* are enriched with sets of Unigenes associated with specific pharmaceutically important metabolic pathways and possess unique medicinal properties [67]. Also, the candidate genes involved in the biosynthesis of benzoic acid and ephedrine in* Pinellia ternata* had been studied and identified by de novo sequencing and transcriptome analysis [54].

### 2.3. Cultivation and Breeding of Chinese Medicinal Herbs

Over a long period time, the cultivation of Chinese medicinal herbs is still along the traditional extensive management mode with low planting management: the seeds and seedlings are reproduction or retention by farmers themselves, so the phenomenon of gene isolation, degeneration, and poor disease resistance is very serious; due to the long time extensive planting, the phenomenon is more serious especially in some local varieties; these problems are often covered in the appearance that “Chinese medicinal herbs are difficult to grow” and not recognized. Therefore, using the modern transcriptional technology to study the metabolism pathway and regulation mechanism in the stage of seed germination is helpful for genetic breeding and making the key influencing factors clear in breeding traditional Chinese medicine. Ephedra as one traditional Chinese herbal medicine has a history of more than four thousand years. However, the problems of low germination rate, long breeding period, and low seedling survival rate always existed. Deng et al. (2015) studied the seed transcriptome of* Ephedra przewalskii* Stapf and found that a total of 16,748 coding genes were involved in 125 metabolic process during seed germination, where metabolic pathways (metabolic pathways) were involved in 3,768 genes, accounting for 22.5% of all encoding genes; then the biosynthesis of the secondary metabolites was involved in 1,888 encoding genes, accounting for 11.27% of all encoding genes; the plant hormone signal transduction accounted for 4.54% (761 encoding genes) [68]. In* Andrographis paniculata* as a large Chinese herbal medicine, there exist some limitations or barriers for crossability within AP accessions, and the problem is solved by using a combination of crossbreeding and genomic data method [69]. Wei et al. (2017) for the first time profiled transcriptome globally in* Loranthus* seeds and studied the genes regulations of* Loranthus* seeds in response to water loss, which make a new understanding of drought tolerance and germination of seeds [64]. The data above laid the foundation for further seedling research.

### 2.4. The Pharmacodynamic Mechanism of Traditional Chinese Medicine

In a large number of Chinese herbal medicines, different parts of the same plant can be classified as different Chinese herbal medicines or pieces, and their pharmacological effects are different. The basis of different parts with different efficacy should be caused by difference chemical composition. And the essence is the difference of gene regulatory network of plant metabolism. Therefore, it is of great significance to research the transcriptome and to reveal the different pharmacological mechanisms for the modernization of traditional Chinese medicine. Lotus plumule and lotus leaf are from the same plant, but the efficacies are significantly different. Lotus plumule is cold in property and can soothe nerves and clear away heart fire; lotus leaf is flat in property and can clear away summer-heat and eliminate dampness. Pharmacological aspects also showed that the lotus plumule can be anticardiac arrhythmia, and lotus leaf has obvious pharmacological activities in weight loss and blood fat. Shan studied the basic reason of different pharmacological effects between lotus plumule and lotus leaf. The study found that the different effects were caused by differences in the chemical composition. The bisbenzylisoquinoline and aporphine alkaloids were the major differences in the composition of the two parts. The different expression pattern of NnCYP80A and NnCYP80 in lotus was suggested to be an important factor that leads to the differentiation of components and finally affected the efficacy [70]. This study provided effective evidences for the elaboration of different pharmacological mechanisms of traditional Chinese medicine, as well as a breakthrough in the study of the mechanism of traditional Chinese medicine.

### 2.5. The Syndrome Differentiation and Treatment of Chinese Medicine

Syndrome differentiation and treatment as the main method of clinical diagnosis and treatment of traditional Chinese medicine is the essence of traditional Chinese medicine. Because determining the TCM symptoms often lacks an objective standard in a deep level, it is difficult to achieve the objective quantitatively in real sense. Both traditional Chinese medicine and the transcription group focus on integrity, so the introduction of transcriptional group is conducive to the comprehensive treatment of TCM diagnosis and treatment. The transcriptome of kidney deficiency syndrome based on the 3 kinds of diseases (impotence, chronic nephritis, and diabetic nephropathy) was analyzed, it was found that there were 332 different expressed genes, and the common signaling pathways associated with kidney yang deficiency syndrome were 37 [71]. Comparing the leukocyte gene expression difference of liver kidney yin deficiency, blood stagnation, and spleen deficiency syndrome through the analysis of chronic hepatitis B caused by liver and kidney yin deficiency syndrome and liver-stagnation and spleen deficiency syndrome by gene chip technology, Guan and Su found that these two syndromes have significant differences between gene expressions. The study of these differences provides a theoretical basis for the view of “the same syndrome for different disease” in traditional Chinese medicine [72].

In TCM syndrome differentiation and treatment system, syndrome differentiation is very important, but to understand Chinese patent medicine mechanism of action is more important. Huang et al. by gene chip combined with RT-PCR method discovered that Jinlong capsule plays anti-brain-tumor effect maybe through upregulating VNN1 [73]. In the study of tumor metastasis related gene expression resulting from Xiaoliu Baofei Pill, Duan et al. found that more than 30 genes were upregulated more than 1.5 times, suggesting that the regulation of traditional Chinese medicine on lung cancer metastasis is multilevel and multitarget [74]. Xie et al. studied the effect of Bu-Shen-Yi-Gan-Huo-Xue decoction on gene expression of mouse bone marrow stromal cells into cartilage differentiation and found that the two important related genes Eefmp1 and Mmp3 had significant differences in expression of cartilage metabolism, indicating that the mechanism of Bu-Shen-Yi-Gan-Huo-Xue decoction in the treatment of osteoarthritis may be related to the pathway mediated by EFEMP1 and MMP3 [75].

### 2.6. Other Researches

The transcriptome research combined with biochemical experimental data can clarify the mechanism of the active components and better explain the profound connotation of the theory of traditional Chinese medicine from the level of gene expression. For example, American ginseng transcriptome studies combined with experiment induced by methyl jasmonate and real-time PCR experiment identified 5 candidate genes involved in the synthesis of ginsenoside [7]. The study of* Lonicera Japonica* transcriptome combined with GC-MS and HPLC technology established the relationship between gene expression amount and the content of active substances [32]. Graham et al. found 3 QTLs loci by analyzing the genotype data and the phenotypic artemisinin concentration [76].

## 3. Conclusion

The transcription group can provide the information of gene expression regulation system (noncoding RNA), all functional protein sequence, and protein interaction [77]. The establishment of transcriptome constitutes a large gene expression information platform, which can be used to study the molecular mechanism of gene expression and regulation of almost all the organisms in the phenomenon of life. Because the transcriptome only determines the expression sequence, there are no large numbers of redundant and repetitive sequences in the genome. Therefore, the study of transcription group sequencing is more simple and practical than the genome sequencing, showing the incomparable superiority [38, 78, 79]. In addition, PacBio has a huge advantage without interruption and assembling, so the Iso-seq (the full-length transcriptome) technology, as a new technology, has been widely used in the traditional Chinese medicine [80, 81]. Synthesizing all the above, transcriptome sequencing encompasses a wide variety of applications from simple mRNA profiling to discovery and analysis of the entire transcriptome, including both coding mRNA and noncoding RNA. Furthermore, transcriptome sequencing can be utilized to analyze transcriptome profiles and deliver unbiased information. Subsequently, transcriptome analysis can identify genetic function in cells and tissues and is important for understanding the development of diseases.

Although the research of Chinese medicine transcription has made great progress, at the same time, we should also be aware of some problems. First, the action mechanism of traditional Chinese medicine is multicomponents and multitargets; the pharmacological interaction cross constitutes a complex pharmacology network. Therefore, only studying some chemical composition or certain types of ingredients to elucidate the pharmacology of traditional Chinese medicine is still one-sided. Second, there are many secondary metabolites in traditional Chinese medicine, and the genome is relatively large, which leads to the lack of genome sequencing or complete genome data and brings some difficulties for the data splicing and annotation. Third, the harvest of Chinese traditional medicine pays attention to the right place and right time and one false step in time will make a great difference in efficacy, such as capillary wormwood herb in traditional Chinese medicine, as the saying goes “Herba* Artemisia scoparia* in March, wormwood in April, cut as firewood in May”; namely, in the 3rd lunar month, the capillary wormwood herb has excellent efficacy, with the effect of clearing heat and removing dampness, benefiting gallbladder jaundice, and being diuretic and detoxifying; in April, capillary wormwood herb lost medicinal value and slowly grew into the white* Artemisia*; this is the best time to eat; and in May, the medicinal and edible values have been lost, only “cut as firewood.” Transcriptome sequencing is an immediate expression of plants, so it has a strict requirement for sampling time.

The concept of “omics” in the transcriptome is consistent with the idea of “whole” in Chinese medicine. Along with the continuous progress of sequencing technology, new technology and new method of proteomics, metabolomics, and network pharmacology, the modernization of traditional Chinese medicine will be developed rapidly.

## Figures and Tables

**Table 1:** Comparison of three generations of sequencing technique.

Sequencing technique	Sequencing platforms	Company	Methods and enzyme	Sequencing length	Output/cycle	Time/cycle	Main error
The first-generation sequencing	Sanger/ABI3730 DNA Analyzer	Applied Biosystems	Sanger sequencing/DNA polymerase	600–1,000 bp	56–96 Kb	0.5–3 h	

The second-generation sequencing	454/GS FLX Titanium Series	Roche	Pyrosequencing/DNA polymerase	400–700 bp	400–700 Mb	10–23 h	Insertion/deletion
	Solexa/Illumina Genome Analyzer/HiSeq 2000/HiSeq 2500/HiSeq 4000/HiSeq X10/NovoSeq	Illumina	Sequencing by synthesis (SBS)/DNA polymerase	2*∗*125 bp (high output)/2*∗*150 bp (rapid run)	900 Gb–1 Tb (paired)/150–180 Gb (paired)	6 d/40 h	Replacement
	SOLiD/SOLiD 3 system	Applied Biosystems	Sequencing by ligation (SBL)/DNA ligase	2*∗*60 bp	90–100 Gb	6-7 d	Replacement

The third-generation sequencing	Heliscope/Helicos Genetic Analysis System	Helicos	SBS/DNA polymerase	30–55 bp	21–35 Gb	8 d	Replacement
	Pacbio RS II/Pacbio Sequel	Pacific Biosciences	SBS/DNA polymerase	1.0–1.5*∗*10^4^ bp	350 Gb-1 Tb	0.5–4 h	
	Nanopore single molecule	Oxford Nanopore	Sequencing by electrical signal/exonuclease	2–5*∗*10^3^ bp	20 Mb–200 Mb	50 h	

**Table 2:** The transcriptome of medicinal plants.

Traditional Chinese medicine name	Sequencing time	Sequencing country	Sequencing platforms
*Artemisia annua* [26]	2009	China	GS FLX™ System
*Epimedium sagittatum* [27]	2010	China	GS FLX System
*Glycyrrhiza uralensis* [12]	2010	China	GS FLX System
*Panax quinquefolius* [11]	2010	China	GS FLX Titanium System
*Salvia miltiorrhiza* [6]	2010	China	GS FLX Titanium System
*Cervus nippon* [28]	2011	China	Illumina HiSeq™ 2000
*C. nippon* [29]	2011	China	Illumina HiSeq 2000
*Panax ginseng* [30]	2011	China	GS FLX Titanium System
*Siraitia grosvenorii* [31]	2011	China	Illumina GA II platform
*Lonicera japonica* [32]	2012	China	Illumina GA II platform
*Carthamus tinctorius* L [17]	2012	China	Illumina HiSeq 2000
*Picrorhiza kurroa* [33]	2012	India	Illumina HiSeq 2000
*Lilium regale* [34]	2012	Netherlands	GS FLX Titanium System
*Polygonum cuspidatum* [35]	2012	China	Illumina HiSeq 2000
*Eucommia ulmoides* [36]	2012	China	Illumina HiSeq 2000
*Cemw elaphus* [37]	2012	China	Illumina HiSeq 2000
*Nelumbo nucifera* Gaertn. [38]	2013	USA	Illumina HiSeq 2000
*L. japonica* [39]	2013	China	GS FLX System
*Aquilaria sinensis* [40]	2013	China	Illumina HiSeq 2000
*Amomum villosum* Lour. [41]	2014	China	Illumina HiSeq 2000
*Fallopia multiflora* [42]	2014	China	Illumina HiSeq 2000
*Conyza blinii* H.Lév. [43]	2015	China	Illumina HiSeq 2500
*Glycyrrhiza uralensis* Fisch. [44]	2015	China	Illumina HiSeq 2500
*Gynostemma pentaphyllum* (Thunb.) Makin [45]	2015	China	Illumina HiSeq 2000
*Erigeron breviscapus.* [46]	2015	China	Illumina HiSeq 2000
*Polygala tenuifolia* [47]	2015	China	Illumina HiSeq 2000
*Xanthium strumarium* L. [48]	2015	China	Illumina HiSeq platform
*Lonicera macranthoides *Hand.-Mazz. [49]	2015	China	Illumina HiSeq 2000
*Lepidium apetalum *Willd. [16]	2016	China	Illumina HiSeq 2000
*Andrographis paniculata* [50]	2016	India	Illumina HiSeq 2000
*Platycodon grandiflorus* [21]	2016	Japan	Illumina HiSeq platform
*Achyranthes bidentata* Bl. [51]	2016	China	Illumina HiSeq 2500
*Forsythia koreana* [52]	2016	Japan	Illumina HiSeq 1500
*Picrorhiza kurroa* [23]	2016	India	Illumina HiSeq platform
*Ephedra sinica* [53]	2016	Japan	Illumina Genome Analyzer IIx
*Pinellia ternata* [54]	2016	China	Illumina HiSeq 2000
*Swertia japonica* [55]	2016	Japan	Illumina HiSeq platform
*Anemone flaccida* [56]	2016	China	Illumina HiSeq 2000
*Atractylodes lancea* [57]	2016	China	Illumina HiSeq platform
*Plantago ovata* [58]	2016	India	Illumina Genome Analyzer II
*Eleutherococcus senticosus* Maxim. [59]	2016	China	Illumina HiSeq platform
*Corydalis* (C.) [60]	2016	China	Illumina HiSeq 2000
*Dendrobium nobile* Lindl. [19]	2017	China	Illumina HiSeq 4000
*Dendrobium officinale* [20]	2017	China	Illumina HiSeq 2500
*Dracocephalum tanguticum* [61]	2017	China	Illumina HiSeq 4000
*Swertia mussotii* [22]	2017	China	Illumina HiSeq 2000
*Elettaria cardamomum* (L.) Maton. [62]	2017	India	Ion Proton sequencer
*Physalis alkekengi* [63]	2017	Japan	Illumina HiSeq 2000
*Physalis peruviana* [63]	2017	Japan	Illumina HiSeq 2000
*Taxillusi chinensis* (DC.) Danser [64]	2017	China	Illumina HiSeq 2000
*Cassia angustifolia* Vahl. [65]	2017	India	Illumina MiSeq platform
